# A Recombinant BCG Vaccine Is Safe and Immunogenic in Neonatal Calves and Reduces the Clinical Disease Caused by the Respiratory Syncytial Virus

**DOI:** 10.3389/fimmu.2021.664212

**Published:** 2021-04-26

**Authors:** Fabián E. Díaz, Mariana Guerra-Maupome, Paiton O. McDonald, Daniela Rivera-Pérez, Alexis M. Kalergis, Jodi L. McGill

**Affiliations:** ^1^ Millennium Institute on Immunology and Immunotherapy, Departamento de Genética Molecular y Microbiología, Facultad de Ciencias Biológicas, Pontificia Universidad Católica de Chile, Santiago, Chile; ^2^ Department of Veterinary Microbiology and Preventative Medicine, Iowa State University, Ames, IA, United States; ^3^ Departamento de Endocrinología, Facultad de Medicina, Pontificia Universidad Católica de Chile, Santiago, Chile

**Keywords:** RSV, neonatal calf model, vaccines, BCG, recombinant BCG

## Abstract

The human respiratory syncytial virus (hRSV) constitutes a major health burden, causing millions of hospitalizations in children under five years old worldwide due to acute lower respiratory tract infections. Despite decades of research, licensed vaccines to prevent hRSV are not available. Development of vaccines against hRSV targeting young infants requires ruling out potential vaccine-enhanced disease presentations. To achieve this goal, vaccine testing in proper animal models is essential. A recombinant BCG vaccine that expresses the Nucleoprotein of hRSV (rBCG-N-hRSV) protects mice against hRSV infection, eliciting humoral and cellular immune protection. Further, this vaccine was shown to be safe and immunogenic in human adult volunteers. Here, we evaluated the safety, immunogenicity, and protective efficacy of the rBCG-N-hRSV vaccine in a neonatal bovine RSV calf infection model. Newborn, colostrum-replete Holstein calves were either vaccinated with rBCG-N-hRSV, WT-BCG, or left unvaccinated, and then inoculated *via* aerosol challenge with bRSV strain 375. Vaccination with rBCG-N-hRSV was safe and well-tolerated, with no systemic adverse effects. There was no evidence of vaccine-enhanced disease following bRSV challenge of rBCG-N-hRSV vaccinated animals, suggesting that the vaccine is safe for use in neonates. Vaccination increased virus-specific IgA and virus-neutralization activity in nasal fluid and increased the proliferation of virus- and BCG-specific CD4+ and CD8+ T cells in PBMCs and lymph nodes at 7dpi. Furthermore, rBCG-N-hRSV vaccinated calves developed reduced clinical disease as compared to unvaccinated control calves, although neither pathology nor viral burden were significantly reduced in the lungs. These results suggest that the rBCG-N-hRSV vaccine is safe in neonatal calves and induces protective humoral and cellular immunity against this respiratory virus. These data from a newborn animal model provide further support to the notion that this vaccine approach could be considered as a candidate for infant immunization against RSV.

## Introduction

The human Respiratory Syncytial Virus (hRSV) is the leading etiological agent of acute lower respiratory tract infections in infants (1), responsible for an estimated 3.4 million hospitalization episodes in children under 5 years of age each year (2). Clinical disease ranges from mild presentations, including rhinorrhea, coughing, and congestion, to respiratory distress, and life-threatening conditions characterized by alveolitis, bronchiolitis, and pneumonia (3). Importantly, hRSV is a significant cause of mortality in this age group, mostly in developing countries (2). Most children are infected by hRSV during the first three years of life, and reinfections are common (4). Furthermore, severe hRSV disease is a predisposing factor for otitis media (5) and has been associated with later health complications, such as development of asthma and recurrent wheezing (6, 7). Besides, extrapulmonary symptoms, including central nervous system pathology and neurological signs have also been linked to hRSV infections (8–11).

Despite the high health burden due to hRSV, no licensed vaccines are available to reduce or prevent the disease caused by this virus in infants (12). An early trial using a formalin-inactivated RSV vaccine (FI-RSV) led to vaccine enhanced disease (VED) upon natural RSV infection in vaccinated volunteers, instead of generating protective immunity against the virus (13–16). Increased hospitalization rate and two fatalities were observed after this trial. Studies on different animal models have associated VED to a Th2 polarized immune response (17–20), a suppressed cytotoxic lymphocyte (CTL) response (19), and an inadequate antibody response (21, 22). However, the immunological mechanisms have been scarcely addressed in mechanistic studies. An essential role for CD4^+^ T cells, but not eosinophils, has been recently demonstrated in *in vivo* mouse models. Interestingly, CD4+ Th1 subsets appear to be responsible for airway obstruction and weight loss, while Th2 subsets account for mucus hypersecretion and airway hyperreactivity (20). Despite that VED mechanisms are still under discussion, RSV vaccine candidates targeting infant populations require evaluating potential VED manifestations in animal models (23). Importantly, these studies highlight the importance of a balanced cellular immunity to prevent immunopathology.

Along these lines, we have shown that a recombinant *Mycobacterium bovis* Calmette-Guerin (BCG) expressing hRSV Nucleoprotein (N) (rBCG-N-hRSV) primes hRSV-specific CD4^+^ T cells and CD8^+^ CTLs that promote antiviral immunity, reduce neutrophil infiltration, and prevent lung damage in a mouse model of infection (24, 25). This vaccine generates a Th1/Th17 biased repertoire of virus-specific memory T cells that confer long-term immunity against hRSV (24, 25), with early recruitment of IFN-γ producing T cells into the lung (26). Furthermore, mice immunized with this vaccine developed a protective humoral response characterized by an isotype class switching towards IgG2a that correlates with viral clearance (27). Importantly, immunization with rBCG-N-hRSV manufactured under current Good Manufacturing Practices (GMP) is safe in mouse models, and induces no observable adverse effects (25). Moreover, recent phase I clinical trial indicated that intradermal administration of doses up to 1 x 10^5^ CFU of GMP rBCG-N-hRSV is safe in healthy adults (28). Considering the extensively accepted safety and immunogenicity profile of the BCG vaccine in newborns (29), the rBCG-N-hRSV is intended for use on neonates to prevent severe hRSV infection (30). However, since the mouse model is not ideal to rule out the possibility of VED (23, 31, 32), further studies employing suitable animal models are required to determine the safety of the rBCG-N-hRSV in target populations.

Bovine RSV (bRSV) is a significant cause of respiratory disease in cattle worldwide, as an agent of enzootic pneumonia in dairy calves and summer pneumonia in nursing beef calves (33–35). Furthermore, bRSV infection is a predisposing factor to secondary bacterial infection and the development of Bovine Respiratory Disease Complex (33–35). These conditions are highly prevalent and a major cause of mortality, as well as of economic losses due to reduced animal performance and costs associated with treatment and control measures (34, 35). Bovine and human RSV are similar at both genetic and antigenic levels, and calf bRSV infection displays many similarities to hRSV infection in humans, including seasonal periodicity, similar age-related susceptibility, gross and microscopic pathology, and innate and adaptive immune responses (34, 36). Severe RSV infection in infants and calves is characterized by an excessive, rapid neutrophil recruitment into lung, a delayed RSV specific CD8^+^ T cells response, and a strong expression of Th2 cytokines (37–40). Such an unbalanced immune response ultimately leads to lung damage and respiratory deficiency (41, 42). In addition, RSV vaccine development needs to overcome similar challenges in humans and the bovine species, including the need to generate a robust immune response in a young population in presence of maternally derived antibodies (MDA). Moreover, VED has been observed in calves after natural (43, 44) and experimental bRSV infection (45, 46), characterized by a Th2-biased immune response, reduced CD8^+^ T lymphocyte response and decreased IFN-γ production (47).

The neonatal calf model is a relevant model for the infant immune system and has been extensively used to study antiviral and therapeutic compounds, and vaccines, including preclinical evaluation of candidate hRSV vaccines that contain proteins that are conserved between hRSV and bRSV (31, 35). Thus, RSV vaccine evaluation in a neonatal calf model might provide useful information for the study of vaccine candidates for infants (23). Here, we evaluated the safety, immunogenicity, and protective efficacy of a GMP rBCG-N-hRSV vaccine (25) in a neonatal bRSV calf infection model. Our results show that vaccination with rBCG-N-hRSV is safe, immunogenic, and partially protective in neonatal calves with MDA. We observed no systemic adverse reactions to the vaccine, and calves developed only minor and resolving vaccine-site reactions following rBCG-N-hRSV immunization. Calves vaccinated with rBCG-N-hRSV mounted virus-specific cellular and humoral immune responses as shown by increased virus-specific IgA and virus-neutralization activity and increased proliferative responses by CD4^+^ and CD8^+^ T cells. Further, rBCG-N-hRSV vaccinated calves developed significantly reduced clinical disease as compared to unvaccinated control calves; however, we did not observe differences in lung pathology or viral replication between vaccinates and controls. Importantly, we observed no evidence of VED following bRSV challenge of rBCG-N-hRSV vaccinated animals, suggesting that the vaccine could be further evaluated for safety and efficacy in neonates.

## Material and Methods

### Animals and Housing

Newborn (2-4 days of age), colostrum-replete, Holstein bull calves were enrolled in the study. The calves were obtained from a local dairy farm free of bovine Tuberculosis, bovine viral diarrhea virus (BVDV) and bRSV. The animals were housed in BSL-2 climate-controlled environment rooms, at the Livestock Infectious Disease Isolation Facility (LIDIF), Iowa State University. Calves were fed commercial milk replacer and later starter concentrates and hay. Water was provided *ad libitum*. Animals were under supervision of a veterinarian throughout the entire study. All animal procedures were conducted in strict accordance with federal and institutional guidelines and were approved by the Iowa State University Institutional Animal Care and Use Committee (IACUC-18-232) and Institutional Biosafety Committee (IBC-18-076).

### Vaccines

The rBCG-N-hRSV (Danish 1331 strain) vaccine (24, 25) used in both studies was produced under current Good Manufacturing Practices (cGMP) standards at IDT Biologika (Rockville, MD USA). A Wild type (WT) BCG, Danish 1331 strain, was used as immunization control in Study 2.

### Immunization Schemes

**Figure 1:**
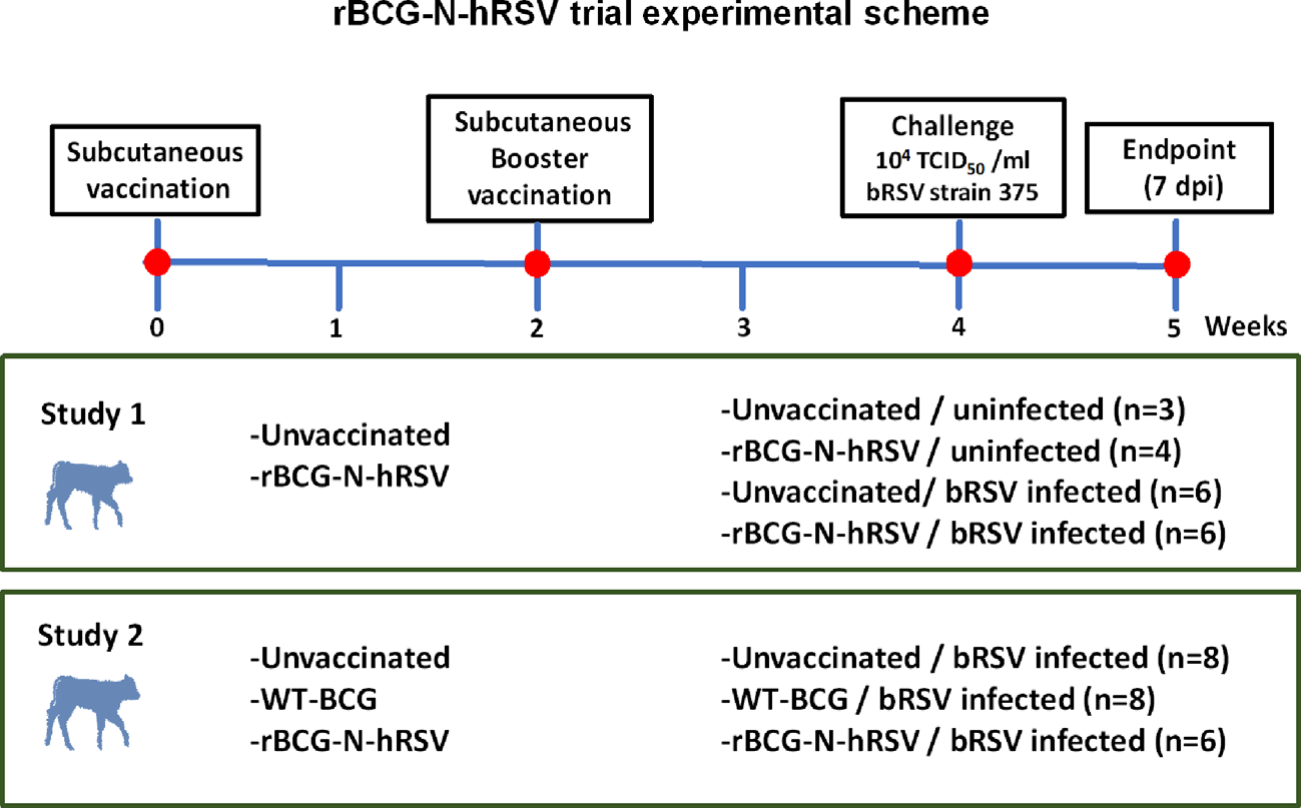
Diagram for the experimental design for studies 1 and 2. Newborn Holstein calves were vaccinated subcutaneously with GMP rBCG-N-hRSV (Studies 1 and 2) or WT BCG (Study 2) and boosted 14 days after prime immunization. Control calves were left unimmunized. After each immunization, animals were monitored for systemic alterations or local reactions to vaccination. Throughout the study, blood was collected weekly from the jugular vein. Fourteen days after the booster, calves were infected with bRSV strain 375 *via* aerosol inoculation. For Study 1, groups were: unimmunized, uninfected (n=3); rBCG-N-hRSV, uninfected (n=4); unvaccinated, bRSV infected (n=6); and rBCG-N-hRSV vaccinated, bRSV infected (n=6). For Study 2, groups were: Unvaccinated (n=8), WT-BCG vaccinated (n=8), and rBCG-N-hRSV vaccinated (n=6), being all animals bRSV-infected Animals were monitored and sampled daily after challenge to obtain blood and nasal fluid samples. All animals were euthanized 7 dpi for pathological evaluation and sampling.

Calves were acclimatized for five days to the study environment prior to vaccination. For Study 1, calves were vaccinated subcutaneously (s.c.) in the right neck with 10^6^ CFU of rBCG-N-hRSV suspended in 500 µl of sterile saline (**Figure 1**). A control group of calves remained unvaccinated. Two weeks after the primary vaccination, a booster vaccination was administered in the right neck with 10^6^ CFU of rBCG-N-hRSV. For Study 2, 8 calves were vaccinated s.c. with 10^6^ CFU of rBCG-N-hRSV, while 8 calves were vaccinated with 10^6^ CFU of WT BCG (**Figure 1**). A control group of 8 calves remained unvaccinated. In both studies, animals were monitored daily for body temperature and injection site reactions for 1 week following each vaccination.

Nasal fluid samples were collected at weekly intervals following vaccination, and on various days post challenge. Sterile 1-2-inch sponges were dampened with 1 mL of sterile saline solution, and then a single square was inserted into the nostril for 5-10 minutes. Then, sponges were removed, placed in a tube, and an additional 1 mL of sterile saline was added. Liquid was recovered from each sponge by squeezing in the barrel of a 5 mL syringe. The resulting nasal fluid was then aliquoted and frozen at -80° C for later analysis. Peripheral blood mononuclear cells (PBMCs) and sera were collected immediately before vaccination, at regular intervals following vaccination, and on days 3 and 7 after challenge.

### Comparative Cervical Tuberculin Tests

A Comparative Cervical Test (CCT) was performed on all animals 10 days after booster immunization. Briefly, 0.1 mL (1 mg/mL concentration) of purified protein derivative (PPD) from M. avium (PPD-A) and of *M. bovis* (PPD-B) were injected in the neck skin of calves three days prior to infection. Then, the reaction size was measured with a caliper and registered as increase of skin thickness for both injection sites, 72h after antigen inoculation and before bRSV challenge. The test and results were performed and interpreted according to the OIE Terrestrial Manual, Eight Edition.

### bRSV Inoculum and Aerosol Challenge Model

BRSV strain 375 was prepared from virus stock re-isolated from the lung of an infected animal and passaged less than 4 times on bovine turbinate (BT) cells. The viral inoculum was determined free of contaminating BVDV by PCR. Two weeks after the booster vaccination, calves were inoculated *via* aerosol challenge with ~10^4^ TCID_50_/mL of bRSV strain 375 as previously described (48).

### Clinical Illness Scoring

For each calf, clinical illness was scored once daily by a trained and blinded observer using an adaptation of the University of Wisconsin Calf Health Respiratory Scoring Chart, originally developed by Dr. Sheila McGuirk (https://www.vetmed.wisc.edu/dms/fapm/fapmtools/8calf/calf_respiratory_scoring_chart.pdf). The scoring chart assigns numbers (0–3) based upon fever and severity of clinical signs that include cough, nasal discharge, eye crusting, and ear position. For our scoring chart we included two additional categories for expiratory effort (0 = no effort to 3 = significant effort) and lung sounds by auscultation (0 = clear, 1 = wheezing and crackling). The scores for each category were totaled to determine the overall clinical score, being 18 the maximum possible score. Any calf with a score equal or over 3 in more than 3 categories for more than 72 hours was euthanized as humane endpoint. One unvaccinated calf was euthanized at 6 days post-infection (dpi) due to severe bRSV during Study 1.

### Necropsy and Pathological Evaluation

Calves were euthanized on 7 dpi by barbiturate overdose. Draining tracheobronchial lymph nodes and lungs of were removed, and dorsal and ventral sides of lungs were photodocumented. Pathological evaluation was performed similar to previous descriptions (48, 49). The extent of gross pneumonic consolidation was evaluated using the scoring system similar to that previously outlined (49). A score of 0 was given to lungs free of lesions; 1 was given to lungs with 1-5% affected; 2 was given for 5-15% affected; 3 with 15-30% affected; 4 to lungs with 30-50% of consolidated tissue; and 5 for lungs >50% affected.

Bronchoalveolar lavage fluid (BAL) was obtained after introducing 500 mL of sterile, ice-cold saline solution through the trachea, and then pouring lavage fluid into a glass bottle. Samples of affected and unaffected lung tissue were collected from eight pre-designated sites for histopathological analysis. Tissues were fixed by immersion in 10% neutral buffered formalin and processed by routine paraffin-embedment and sectioning. Five µm sections were H&E stained. Microscopic lesions were evaluated by a board-certified veterinary pathologist in a blinded manner. The severity of the lung lesions was scored based upon the criteria we have previously established (50, 51).

### Real Time PCR Analyses

For quantification of bRSV NS2 copy number, lung samples from 2 representative gross-lesioned and 2 non-lesioned tissues from each calf were collected and stored in RNAlater (Invitrogen, Life Technologies). RNA was isolated from lung tissue samples using Trizol Reagent (Invitrogen, Life Technologies). Total RNA was placed in Qiagen RNA isolation columns for RNA clean-up and to remove any contaminating DNA using RNAse-free DNase, per the instructions of the manufacturer (Qiagen). For nasal samples, viral RNA was isolated using MagMax Viral RNA Isolation Kit per the manufacturer’s instructions (Applied Biosystem, Life Technologies. The quality and quantity of isolated RNA was verified by QuBit 4 Fluorometer (Thermofisher Scientific), and 500 ng of total RNA were used in each reaction. cDNA synthesis and quantitative rtPCR reactions were carried out using the Taqman RNA-to-CT 1-step kit (Applied Biosystems) per manufacturer’s instructions using the following primers and probes: NS2 forward, 5’-GAACGACAGGCCACATTTA-3’; NS2 reverse, 5’- AGGCATTGGAAATGTACCATA-3’; NS2 probe, 5’-/56-FAM/TGAAGCTAT/ZEN/TGCATAAAGTGGGTAGCACA/3IABkFQ/-3’; RPS9 forward, 5’-GTGAACATCCCGTCCTTCAT-3’; RPS9 reverse, 5’-TCTTGGCGTTCTTCCTCTTC-3’; RPS9 probe, 5’-/56-FAM/AAGTCGATG/ZEN/TGCTTCTGCGAGTCC/3IABkFQ/-3’. The reactions were performed on a ThermoFisher QuantStudio 3 Real-Time PCR machine with the following cycling conditions: 48° C hold for 15 minutes; 95° C hold for 10 minutes; 40 cycles of 95° C for 15 seconds, then 60°C for 1 minute. Standard curves for NS2 and RPS9 genes were run in parallel with test samples, and all standards and test samples were run in triplicate. DNA sequences coding for nucleotides 1-706 of bovine RPS9, and nucleotides 524-1152 of bRSV NS2, both cloned separately into PCR2.1-TOPO vectors, were employed as templates for standard curve construction, respectively. Viral NS2 copy numbers were calculated using standard curves and normalized to RPS9 to correct for differences in lung tissue input.

### Virus Isolation

Samples from lesioned lung tissue were snap-frozen during necropsy and stored at -80° C until use. Nasal swabs were collected from each calf prior to infection and on various days post infection and placed in 500 µl virus transport media. Swabs were vortexed vigorously in the media, removed from the collection tube, and the supernatant was stored at -80° C. Samples were thawed once, and a 200 µl aliquot was removed for qPCR. The remaining volume was used for virus isolation, which were performed as previously described (48).

### Serum and Nasal Fluid Neutralization assays

Serum and nasal fluid samples collected immediately before challenge and at 7dpi were submitted to the Iowa State University Veterinary Diagnostic Laboratory (Ames, Iowa) for evaluation of bRSV-specific neutralization titers.

### Antigen Recall Assays

Bovine peripheral blood was drawn from the jugular vein into syringes containing 2 × acid-citrate-dextrose solution. For isolation of PBMCs, blood was diluted 1:1 in PBS, and centrifuged for buffy coat fractions. Then, those fractions were centrifuged with Histopaque-1077 (Sigma-Aldrich) to obtain isolated PBMCs. Erythrocytes were removed incubating 5 minutes in warm RBC lysis buffer. Finally, cells were washed three times, counted, and resuspended in complete RPMI (cRPMI) composed of RPMI-1640 (Gibco) supplemented with 10% (v/v) heat-inactivated fetal bovine sera (FBS), 2 mM L-glutamine, 1% antibiotic-antimycotic solution, 1% non-essential amino acids 2% essential amino acids, 1% sodium pyruvate, and 50 μM 2-mercaptoethanol (all from Sigma-Aldrich). Besides, BAL samples obtained during necropsies were kept on ice, filtered over sterile gauze, and centrifuged 10 minutes at 200g, 4 °C. Erythrocytes were removed incubating 5 minutes in warm RBC lysis buffer. Also, tracheobronchial lymph nodes were collected, and cells obtained after disaggregating the tissue on cRPMI and passing the cell suspension through a 40 μm cell strainer. For all samples, cells were washed, counted, and resuspended in cRPMI. Then, PBMCs and lymph node cells were resuspended in 1 ml of PBS containing 10 μM of the CellTrace Violet (CTV) stain, and incubated 20 minutes at 37 °C. Labeling was quenched by adding 4 volumes of RPMI, and washed two times with RPMI (Invitrogen, Life Technologies). For all samples, 5x10^6^ cells/mL were plated in round-bottom 96-well plates with 10 µg/mL Purified Protein Derivative from *M. bovis* (PPD-B); a cocktail of 10 µg/mL each recombinant Ag85A and TB10.4 (Lionex GmbH); 10 µg/mL each recombinant N from human RSV (N-hRSV); or a 0.1 MOI of bRSV strain 375. Negative control (mock) wells remained unstimulated. Positive control wells were stimulated with 5 µg/mL Concanavalin A (ConA). Plates were incubated for 6 days at 37° C in a 5% CO_2_ incubator. Then, cell culture supernatants were stored at -80° C, and PBMCs immediately stained. PBMCs were suspended in FACS buffer (10% FBS and 0.02% NA-azide in PBS) and incubated 30 minutes at 4°C with 10 μg/mL of mouse anti-bovine CD4 and 10 μg/mL of mouse anti-bovine CD8α (clones ILA11A & BAQ111A respectively, both from Kingfisher Biotech, Inc). After washing, cells were incubated 25 minutes at 4°C with anti-mouse IgG2a-FITC and anti-mouse IgM-APC, and then fixed with BD FACS lysis buffer (BD Biosciences) for 10 min at RT, washed, and resuspended in FACS buffer. Cells were analyzed using a BD FACS Canto II (BD Biosciences) and FlowJo Software (Treestar). Percentages of cell proliferation were expressed over mock conditions.

### ELISAs

Bovine IL-17A and IFNγ were quantified using commercial bovine kits according to the instructions provided by manufacturer (Kingfisher Biotech, Inc). Indirect ELISAs were used to quantify IgA in the nasal fluid (Studies 1 and 2) and total IgG (Study 1) in serum. For the IgA quantification, 96-well ELISA plates were coated overnight at 4° C with 100 µl/well of bRSV stock (~10^4^ TCID_50_). Negative control wells were coated with 100 µl/well cell culture media prepared from uninfected BT. To disrupt mucus, nasal fluid samples were diluted 1:2 and treated with 10 mM dithiothreitol for 1 hour at 37° C prior to plating. Serum samples were diluted 1:1000. Plates were blocked using 150ul/well of 1% nonfat dry milk in PBS. All samples were plated in duplicates, incubated for 2 hours at RT, and then washed with 200 ul/well of 0.05% Tween 20 in PBS. Then, plates were incubated 1 hour at RT with either Mouse anti-bovine IgA-HRP (Bethyl Laboratories) at 0.5 µg/mL, or mouse anti-bovine IgG-HRP (Bethyl Laboratories) at 0.5 µg/mL. After incubation, plates were washed three times with 200 ul/well of 0.05% Tween 20 in PBS, and then developed using 50ul/well of Pierce 1-Step Ultra TMB Substrate (ThermoScientific Pierce). The reaction was stopped with the addition of 50ul/well 0.2 M H_2_SO_4,_ and plates were read using a 450 nm wavelength, with a 540 nm reference wavelength, using an automated plate reader. For Study 2, bovine IgG1 was quantified using commercial Svanovir BRSV Ab kit (Svanova, Boehringer Ingelheim) according to the instructions provided by the manufacturer. Bovine IgG2 was quantified modifying Svanovir BRSV Ab kit, by incubating with sheep anti-bovine IgG2-HRP (Bethyl Laboratories) instead of provided secondary antibody reagent. All samples were plated in duplicate and included a negative control well.

### Statistical Analyses

For relative gene expression analyses, ΔΔCt values were used to calculate 2^-ΔΔCt^ (52), and results are shown as expression relative to uninfected control samples. Results are expressed as average ± standard error of the mean (SEM). Statistical significance was determined by two-way Analysis of Variance (ANOVA) or two-way ANOVA with repeated measures, followed by Sidak’s multiple comparisons test using GraphPad Prism 7 software (GraphPad Software, Inc).

## Results

### Vaccination With rBCG-N-hRSV in Neonatal Calves Shows a Good Safety Profile

To evaluate the safety of the rBCG-N-hRSV vaccine in a neonatal calf model, animals were monitored daily for body temperature and injection site reactions for one week following each vaccination. Calves were vaccinated with the rBCG-N-hRSV vaccine or WT-BCG at 2-4 days of age and then boosted two weeks later. All animals were monitored daily for body temperature and injection site reactions for one week following each vaccination. During Study 1, minor injection site reactions were observed in 11 of the 12 rBCG-N-hRSV vaccinated calves and included minor swelling and hardening of the vaccination site. Vaccination site reactions resolved within 4-5 days. Following booster vaccination, injection site reactions were observed in all vaccinated calves and included thickening and hardening of the skin surrounding the injection site. Those reactions resolved within 7-10 days after vaccination. In study 2, only one rBCG-N-hRSV vaccinated calf developed minor swelling and hardening of the skin after the first immunization, which resolved within three days. No reactions were observed in WT BCG vaccinated or unvaccinated calves. Furthermore, no significant body temperature changes were observed following booster vaccination in any animal (data not shown).

### Vaccination With rBCG-N-hRSV Ameliorates bRSV Clinical Symptoms Without Signs of Enhanced Disease

**Figure 2:**
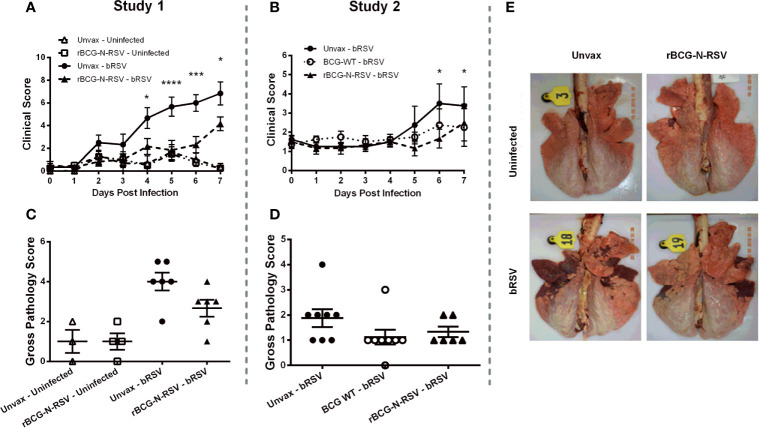
rBCG-N-hRSV vaccination reduces bRSV-associated disease in neonatal calves. Newborn calves were vaccinated with rBCG-N-hRSV (Studies 1 and 2) or WT BCG (Study 2) and boosted 14 days after prime immunization. Fourteen days after the booster, calves were infected with BRSV strain 375 *via* aerosol inoculation. **(A, B)** Clinical Scores. Calves in all four groups were monitored daily by a blinded observer and assigned a clinical score using the criteria outlined in Materials and Methods. Data represented as mean ± SEM. *p<0.05 ***p<0.001 ****p<0.0001 as determined by 2-way ANOVA with repeated measures and Sidak’s multiple comparisons test **(C–E)** Gross Pathology Scores. All animals were humanely euthanized on day 7 post-infection. The extent of gross pneumonic consolidation was evaluated based upon the percent of lung affected (0=free of lesions; 1 = 1-5% affected; 2 = 5-15% affected; 3 = 15-30% affected; 4 = 30-50% affected; 5 = >50% affected). Aggregate gross pathology results from all groups and all animals from Study 1 and 2 are depicted in **(C, D)**, respectively. Data represented as mean ± SEM. No statistically significant differences were observed. **(E)** Representative images from one animal from each group from Study 1.

Two weeks after the booster immunization, calves were challenged *via* aerosol inoculation with 10^4^ TCID_50_ bRSV strain 375. Control calves were not challenged. Following infection, all animals were monitored daily for body temperature and clinical signs, as described in the Materials and Methods section. During study 1, unvaccinated bRSV infected calves displayed significant clinical signs beginning on days 4-5 after infection, which included fever, lethargy, nasal and ocular discharge, dyspnea, and lung sounds (**Figure 2A**). One animal was euthanized on day six after infection due to severe clinical disease. Although calves immunized with rBCG-N-hRSV developed some signs of bRSV infection, disease and clinical scores were significantly reduced on days 4-7 pi, as compared to unvaccinated and infected calves (p<0.05 for day 4 and 8 pi, <0.001 for day 6pi and <0.0001 for day 5 pi) (**Figure 2A**). Unvaccinated and challenged calves presented fever starting day 4 pi, while rBCG-N-hRSV-vaccinated calves had no rise in body temperature (**Supplementary Figure 1A**). During study 2, unvaccinated, bRSV infected calves also developed clinical signs, including fever, lethargy, nasal and ocular discharge, and mild dyspnea (**Figure 2B**). An increase in clinical score on days 6-7 was evidenced in unvaccinated calves when comparing scores at those days with day 0 (pre-challenge) (p<0.05). Some vaccinated calves also developed clinical signs; however, no significant rise in clinical score was observed at any day when compared to day 0. Similarly, no significant rise in clinical score was observed for WT BCG-vaccinated animals. Although some unvaccinated animals had fever, we observed no statistically significant differences in body temperature between vaccinated and unvaccinated animals (**Supplementary Figure 1B**). Importantly, we observed no signs of VED in calves receiving the rBCG-N-hRSV or the WT BCG vaccines.

**Figure 3:**
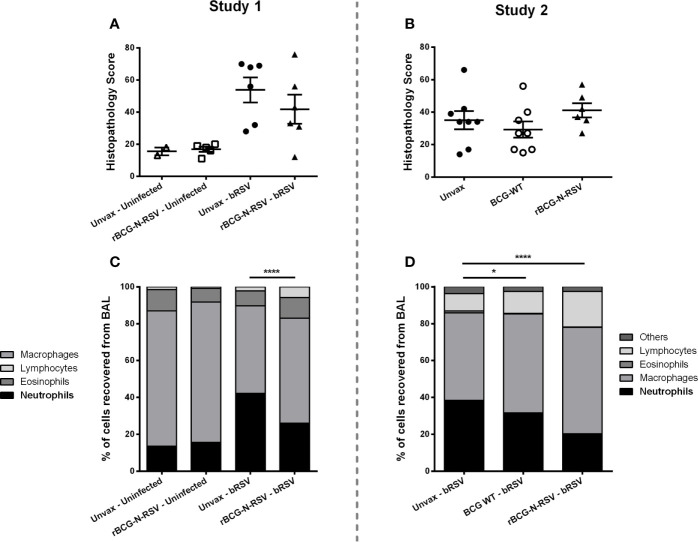
rBCG-N-hRSV vaccination reduces neutrophil BAL infiltration in neonatal calves after bRSV infection. **(A, B)** Lung Histopathology Score. Sections of lungs were collected from 8 predesignated locations and microscopic lesions were evaluated by a pathologist in a blinded manner using a scoring system we have previously described. Aggregate histopathology scores from animals in Study 1 and 2 are depicted in **(A, B)**, respectively. Data represented as mean ± SEM. No statistically significant differences between infected groups were observed. **(C, D)** BAL cells relative frequency. On day 7 post-infection, BAL samples were collected, and cytospins prepared. The cells were differentially stained with Modified Wright stain. The number of neutrophils, macrophages, lymphocytes, and eosinophils were determined by microscopy. Data are depicted as mean relative frequencies of each population. Data represented as mean ± SEM. *p<0.05 ****p<0.0001 for the frequencies of neutrophils as determined by 2-way ANOVA and Sidak’s multiple comparisons test.

To determine the extent of macroscopic lung damage after bRSV infection, calves were euthanized seven days post-infection, and the lungs were evaluated and scored for gross pathology by a blinded veterinary pathologist, as described in the Materials and Methods section. No significant lesions were observed in the lungs of the uninfected control calves in study 1 (**Figures 2C, E**). Calves challenged with bRSV developed evident macroscopic lung pathology, including regional and coalescing areas of lung consolidation (**Figures 2C, E**). The extent of lesions in challenged calves was greater in the Study 1 as compared to Study 2. Although no significant differences in gross lung pathology were observed between unvaccinated control calves and those receiving the rBCG-N-hRSV vaccine, a clear reduction of lung damage was observed for the rBCG-N-hRSV-vaccinated calves in both studies (**Figures 2C, D**). Remarkably, most of the WT BCG-vaccinated animals in study 2 showed very little macroscopic pathology (**Figure 2D**). Additionally, samples of eight predesignated regions of the lung were collected and formalin-fixed during necropsy for histopathological evaluation. Lungs were sectioned and scored by a blinded veterinary pathologist. Few microscopic lesions were observed in the lung tissue samples collected from the uninfected calves (**Figure 3A**). On the other hand, and as expected, calves challenged with bRSV developed extensive histologic lesions, including airway inflammation and necrosis, bronchiolar luminal exudate, leukocyte and lymphocyte infiltration, and pneumocyte hyperplasia. Overall, there were no significant differences in the lung histopathology scores between unvaccinated animals, WT BCG, or rBCG-N-hRSV vaccinated calves infected with bRSV (**Figures 3A, B**).

Severe RSV disease in newborn humans and calves is characterized by bronchointerstitial pneumonia and bronchiolitis, as well as significant airway neutrophil infiltration (32, 34). The local host inflammatory reaction to the infection is a major cause of tissue damage (41, 53–55), with neutrophils pointed out as an important immunopathology source (50, 56). Next, we evaluated whether vaccination with rBCG can modulate neutrophil infiltration at the site of infection. At necropsy, BAL fluid was collected from each animal and cytospin preparations were differentially stained. The relative numbers of neutrophils, macrophages, lymphocytes, and eosinophils were then quantified by microscopy. As expected, bRSV infection increased neutrophil infiltration into the airways, and the frequency of neutrophils was increased in the BAL of challenged animals as compared to uninfected controls (p<0.0001) (**Figure 3C**). However, in both studies, rBCG-N-hRSV-vaccinated calves showed significantly reduced frequencies of neutrophils in the BAL at 7 dpi (p<0.0001) as compared to unvaccinated infected animals (**Figures 3C, D**). Remarkably, a mild, significant reduction in neutrophil frequency was observed for WT BCG-vaccinated animals when compared to unvaccinated animals (p<0.05) (**Figure 3D**). In both studies, a lower relative frequency of macrophages was observed in unvaccinated, challenged animals when compared to vaccinated calves (p<0.05, <0.001 for study 1 and 2, respectively) (**Supplementary Figures 2A, E**). While no differences in relative lymphocyte frequency were observed between any group in Study 1, significantly higher relative lymphocyte counts in rBCG-N-hRSV-vaccinated animals were seen in study 2 as compared to unvaccinated controls (p<0.001) (**Supplementary Figures 2B, F**). Finally, no differences were seen in eosinophil infiltration between groups (**Supplementary Figures 2C, G**).

### Viral Shedding and Viral Lung Loads Are Not Reduced in rBCG-N-hRSV Vaccinated Calves

Nasal swabs and lung tissue were collected and immediately frozen, then processed for virus isolation as previously described (48). As shown in **Table 1**, no virus was isolated from the nasal swabs of any calf prior to challenge. Following bRSV infection, virus was isolated from the nasal swabs of most infected animals throughout the infection period, regardless of vaccination status. For Study 1, bRSV was isolated from lung tissue samples in 6/6 calves in the unvaccinated infected group and from 4/6 rBCG-N-hRSV vaccinated, infected calves on day 7 after infection. Regarding Study 2, the virus was isolated from lung tissue of all unvaccinated and WT BCG-vaccinated animals and in 4/5 rBCG-N-hRSV vaccinated animals (**Table 1**). Quantitative PCR analyses for the bRSV NS2 gene revealed no statistically significant differences between vaccinated and unvaccinated infected calves for the copy number of NS2 in lesioned lung tissue (**Table 1**). Neither virus nor NS2 copies were detected, in uninfected control calves. These results suggest that neither rBCG-N-hRSV nor WT BCG significantly modulate virus replication in the lower and upper respiratory tract during neonatal calf bRSV infection.

**Table 1:** Virus shedding and lung viral loads in the nasal swabs and lungs.

Group		Study 1					Study 2		
		Nasal Swabs				Lung	Nasal Swabs		Lung
		Day 0	Day 2pi	Day 4pi	Day 7pi	Day 7pi	Day 3pi	Day 7pi	Day 7pi
**Unvaccinated - bRSV**	Virus isolation	0/6	6/6	5/6	4/6	6/6	7/7	7/7	7/7
	NS gene copies/10^4^ RPS9 copies		602 (0-2,544)	29,448 (0-164,817)	1,604 (0-4,986)	122 (1-488)	43,560 (0-190,000)	86,189 (0-547,000)	1,427 (0-9,274)
**rBCG-N-hRSV - bRSV**	Virus isolation	0/6	4/6	4/6	3/6	4/6	6/7	6/6	4/5
	NS gene copies/10^4^ RPS9 copies		56 (0-176)	4,684 (0-14,790)	129 (0-449)	73 (1-351)	66,354 (315-210,000)	540,880 (0-2,470,000)	1,335 (15-4,489)
**WT BCG - bRSV**	Virus isolation	–	–	–	–		7/7	7/7	8/8
	NS gene copies/10^4^ RPS9 copies						2,640 (0-12,700)	946,478 (176-4,460,000)	1,069 (0-6,399)

Nasal swabs were collected from each calf prior to infection and on various days post infection. Lesioned lung tissue was collected at necropsy. Samples were divided and analyzed for virus isolation and virus quantification by qPCR for the bRSV NS2 gene. Virus isolations were performed as previously described Viral NS2 copy numbers were calculated using standard curves. For nasal swabs, 500 ng of isolated RNA were used in the qPCR reactions. Lung tissue were normalized to the housekeeping gene, RPS9, to correct for differences in input material. NS2 copies presented as mean (range) of each group. Neither virus nor NS2 copies were isolated or detected, respectively, in uninfected control calves. No significant differences were observed between unvaccinated, WT BCG and rBCG-N-hRSV vaccinated animals as determined by RM 2-way ANOVA and Sidak’s multiple comparisons test.

### Vaccination With rBCG-N-hRSV Induces Antigen-Specific CD4^+^ and CD8^+^ T Cells Secreting Th1/Th17 Cytokines Upon bRSV Challenge

**Figure 4:**
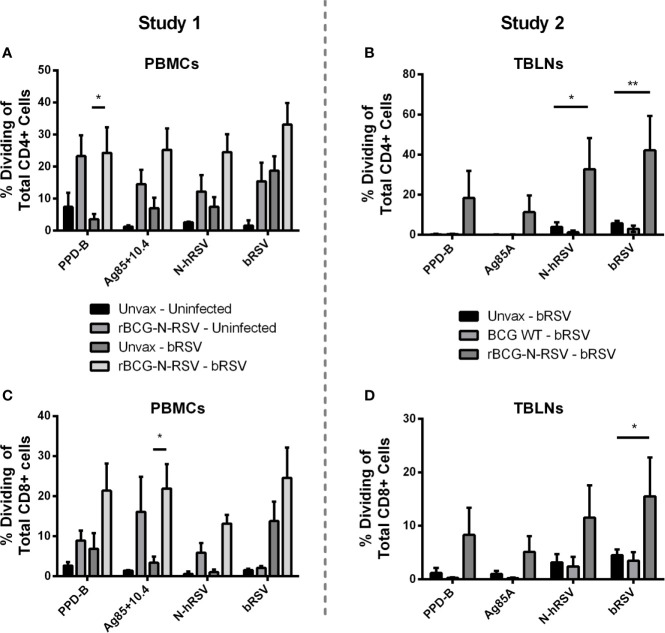
Vaccination with rBCG-N-hRSV elicits antigen-specific CD4 and CD8 T cell proliferative responses. **(A, C)** Study 1 PBMCs and **(B, D)** Study 2 Tracheobronchial lymph node cells (TBLNs) were isolated on day 7 after infection, labeled with Cell Trace Violet, and restimulated *in vitro* with PPD-B, Ag85A/TB10.4, N-hRSV or bRSV strain 375. Mock stimulated cultures were used as negative controls. ConA stimulated cultures were used as positive controls (not shown). Six days later, **(A, B)** CD4^+^ T and **(C, D)** CD8^+^ T cell proliferation, as measured by dilution of the Cell Trace dye, was analyzed by flow cytometry. Data represented as mean ± SEM *p<0.05 **p<0.01 as determined by 2-way ANOVA and Sidak’s multiple comparisons test.

**Figure 5:**
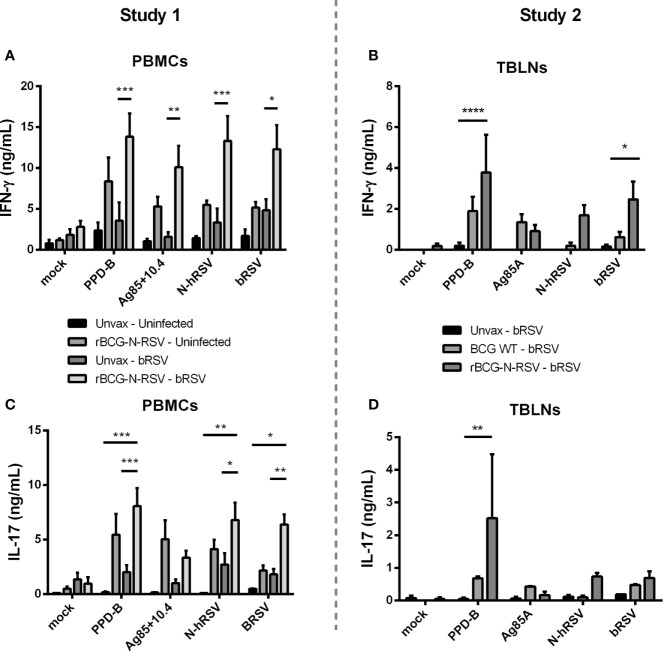
Vaccination with rBCG-N-hRSV increases virus and BCG-specific IFN-γ and IL-17 secretion in PBMC cultures. **(A, C)** Study 1 PBMCs and **(B, D)** Study 2 Tracheobronchial lymph node cells (TBLNs) were isolated on day 7 post-infection, labeled with Cell Trace Violet, and restimulated *in vitro* with PPD-B, Ag85A/TB10.4, N-hRSV or bRSV strain 375. Mock stimulated cultures were used as negative controls. ConA stimulated cultures were used as positive controls (not shown). Six days later, cell culture supernatants were analyzed for bovine IFN-γ and IL-17A secretion using commercial ELISA kits. *p < 0.05 **p < 0.01 ***p < 0.001 ****p < 0.0001 as determined by Two-way ANOVA and Sidak’s multiple comparisons test. Data represented as mean ± SEM.

To evaluate the effect of rBCG-N-hRSV vaccination on adaptive cellular immunity after calf bRSV infection, PBMCs and lymph node cell cultures were collected on day seven pi, labeled with CTV stain, stimulated with viral or mycobacterial antigens for six days, and then analyzed by flow cytometry. Antigen-specific CD4^+^ and CD8^+^ cells were identified by CTV dilution after proliferation in response to viral or mycobacterial antigens. Background (mock) proliferation was subtracted from all values, and results represent change over mock (**Supplementary Figure 3**). For PBMCs obtained from Study 1, we observed no statistically significant differences in CD4^+^ or CD8^+^ T cell proliferation between both infected groups when stimulated with viral antigens. Although trends to increased proliferative responses were observed for CD4^+^ T (**Figure 4A**) and CD8^+^ T (**Figure 4C**) in response to N-hRSV and bRSV in the vaccinated/bRSV infected calves, increased T cell proliferation was also observed in samples from vaccinated uninfected animals (**Figures 4A, C**). Besides, CD4^+^ T cells from calves receiving the rBCG-N-hRSV vaccine responded robustly to both PPD-B and the Ag85A/TB10.4 antigen cocktail (**Figure 4A**). As expected, CD4^+^ T cells from the unvaccinated/bRSV infected calves did not divide in response to stimulation with PPD-B or Ag85A/TB10.4 (**Figure 4A**). Similar trends were observed for CD8^+^ T cells, with the highest responses observed in samples from the rBCG-N-hRSV-vaccinated and challenged calves (**Figure 4C**). To evaluate if rBCG-N-hRSV vaccination promotes a Th1/Th17 phenotype in calves, as reported in the murine and human studies (24, 25, 28), cell culture supernatants from the stimulated PBMCs were analyzed by ELISA for bovine IFN-γ and IL-17A. Compared to the PBMCs from unvaccinated/bRSV infected animals, rBCG-N-hRSV-vaccinated/bRSV infected animals mounted a significant IFNγ (**Figure 5A**) and IL-17A (**Figure 5C**) response to both BCG- and RSV-associated antigens including PPD-B, Ag85A/TB10.4, N-hRSV, and BRSV strain 375. Some cytokine responses were seen in unvaccinated, bRSV infected calves, although those responses were not statistically significant when compared to unstimulated control wells. All samples produced robust amounts of IFNγ and IL-17A in response to conA, which was used as a positive control (not shown). Additionally, BAL samples were stimulated with mycobacterial and viral antigens for 6 days and supernatants analyzed by ELISA for bovine IFN-γ and IL-17A. As shown in **Supplementary Figure 4**, rBCG-N-hRSV-vaccinated, infected animals produced significantly higher IFN-γ in comparison to unvaccinated, infected animals (p<0.05), however, the enhanced IL-17A levels in response to PPD-B and AG85A/TB10.4 did not reach statistical significance. Both infected groups produced similar levels of IFN-γ and IL-17A in response to viral antigens at 7 dpi.

For the second study, we analyzed proliferative responses of CD4^+^ and CD8^+^ T cells, and IFNγ and IL-17A secretion in tracheobronchial lymph node cell cultures, performing assays as described above. CD4^+^ T cells from vaccinated calves showed robust proliferation after recall stimulation with N-hRSV (p<0.05) or bRSV (p<0.01) in comparison to the unvaccinated control group (**Figure 4B**). Differences in proliferation after PPD-B or Ag85 stimulation between vaccinated and unvaccinated control groups were not statistically significant. As expected, CD8^+^ T cell responses were also robustly induced after recall stimulation with bRSV in lymph node cells from rBCG-N-hRSV-vaccinated animals (p<0.05) but not unvaccinated or WT BCG-vaccinated control animals (**Figure 4D**). Although statistically significant differences were not found when comparing rBCG-N-hRSV-vaccinated calves to unvaccinated controls, a higher proliferation of CD8^+^ T cells was observed in rBCG-N-hRSV-vaccinated calves upon recall PPD-B and N-hRSV stimulation (**Figure 4D**). Importantly, cells from all calves included in data analyses showed a robust proliferative response to Con A positive control (not shown). Furthermore, rBCG-N-hRSV-vaccinated animals also showed a significantly higher IFN- γ response to PPD-B (p<0.0001) and to bRSV (p<0.05) stimulation (**Figure 5B**), which was not seen on WT BCG and unvaccinated animals (**Figure 5B**). A similar trend was observed in N-hRSV-stimulated wells, although differences did not reach statistical significance (**Figure 5B**). Regarding IL-17A, a robust response to PPD-B (p<0.01) was seen only in rBCG-N-hRSV-vaccinated calves (**Figure 5D**). In summary, these results indicate that vaccination with rBCG-N-hRSV induce antigen-specific CD4^+^ and CD8^+^ T cells associated with a Th1/Th17 secretory phenotype upon bRSV infection in neonatal calves with MDA.

### Humoral Immune Responses to Vaccination and bRSV Challenge in Neonatal Calves

**Figure 6:**
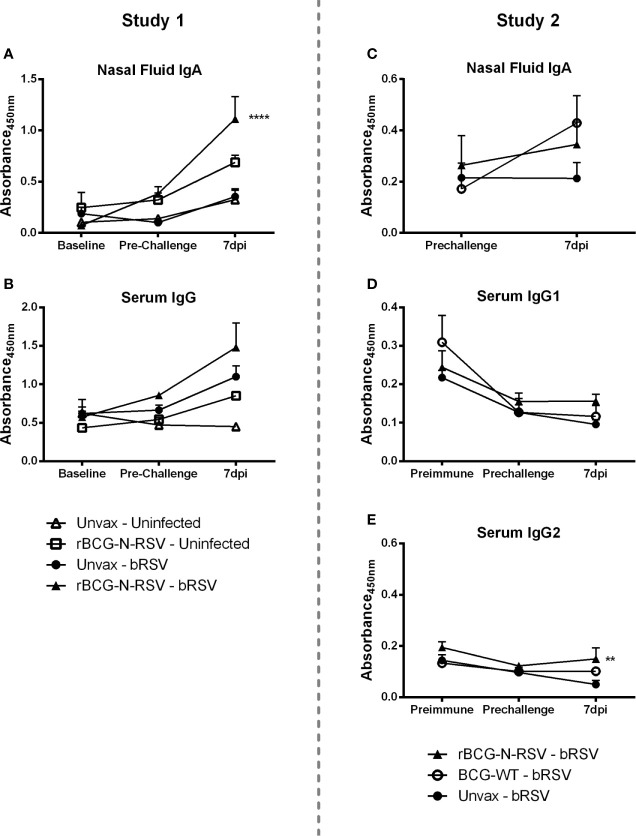
rBCG-N-hRSV vaccinated calves mount an amnestic virus-specific IgA and IgG2 response in the respiratory tract and peripheral blood, respectively. **(A, C)** Nasal fluid samples were collected and analyzed for bRSV-specific IgA by indirect ELISA prior to immunization (baseline), at pre-challenge, and on day 7 post-infection. Data represented as mean ± SEM. No significant differences were detected in IgA levels at baseline and pre-challenge between any group. ****p<0.0001 between rBCG-N-hRSV-vaccinated – bRSV and unvaccinated – bRSV groups on day 7 post infection as determined by 2-way ANOVA followed by Sidak’s multiple comparisons test. **(C, E)** Serum samples were collected and analyzed for IgG (**B**, Study 1), IgG1, and IgG2 (**D, E**, Study 2). **p < 0.01 between rBCG-N-hRSV-vaccinated – bRSV and unvaccinated – bRSV groups on day 7 post-infection as determined by 2-way ANOVA followed by Sidak’s multiple comparisons test.

To analyze the humoral immune responses induced by rBCG-N-hRSV vaccination, nasal fluid and serum samples were collected at several time points to analyze virus-specific IgA and IgG, respectively. In the Study 1, virus-specific IgA was undetectable in the nasal fluid from any group at baseline (prior to vaccination) or immediately prior to infection (**Figure 6A**). Virus-specific IgA remained below the limit of detection in uninfected control calves throughout the study. By day 7 post-infection, unvaccinated infected calves were beginning to show a virus-specific IgA response in the nasal tract. However, this increase was not statistically significant as compared to the pre-challenge values or to uninfected controls. On the other hand, rBCG-N-hRSV-vaccinated calves developed an anamnestic virus-specific IgA response in the respiratory tract, evidenced by significantly higher levels of virus-specific IgA in the nasal fluid as compared to all other groups (p<0.0001) (**Figure 6A**). Analyses of total virus-specific IgG revealed a similar trend, but the differences between groups were not statistically significant (**Figure 6B**). In Study 2, an increase in IgA levels was observed in WT BCG- and rBCG-N-hRSV-vaccinated calves but not in unvaccinated controls when comparing pre-infection levels to 7dpi, however, this difference was not statistically significant (**Figure 6C**). Comparisons between groups at 7dpi showed no differences in IgA levels. In Study 2, virus-specific IgG1 and IgG2 were measured. No differences between groups at any time point were observed for virus-specific IgG1 serum levels. It is noteworthy that IgG1 levels were higher in the unvaccinated and WT BCG-vaccinated group at baseline, as compared to later time points, and as expected, no rise in IgG1 serum levels was seen in those groups at 7dpi (**Figure 6D**). Although a similar trend was observed in rBCG-N-hRSV-vaccinated calves, no significant changes between any time point were observed in this group. In contrast, rBCG-N-hRSV-vaccinated calves showed significantly higher serum levels of IgG2 at 7dpi (p<0.01) when compared to unvaccinated animals, whereas unvaccinated calves showed a decreasing trend in IgG2 levels when comparing baseline to 7 dpi levels (**Figure 6E**). Importantly, animals in both studies were colostrum replete, which is likely the main factor contributing to the higher IgG1 and IgG2 serum levels at baseline.

Virus neutralization assays revealed that all groups, regardless of treatment, had similar pre-challenge titers of neutralizing antibodies in nasal secretions (**Table 2**). however, neutralizing titers (NTs) increased significantly in the nasal fluid samples from rBCG-N-hRSV-vaccinated animals following bRSV challenge in both studies (p<0.05) (**Table 2**). Interestingly, WT BCG vaccinated calves also had higher NTs from nasal samples when compared un unvaccinated animals (p<0.01). On the other hand, a trend in the increase in NTs in the serum of the rBCG-N-hRSV vaccinated calves was observed in Study 1, but this increase was not statistically significant, which is along the lines with virus-specific IgG serum levels. No differences in serum NTs were observed in study 2 when comparing different groups or time points.

**Table 2:** Virus neutralization titers.

Group mean NT (range)	Nasal Fluid NTs				Serum NTs				
	Study 1		Study 2		Study 1		Study 2		
	Day 0	7dpi	Day 0	7dpi	Day 0	7 dpi	Baseline	Day 0	7 dpi
**Unvaccinated - Uninfected**	3 (2-4)	2 (2)	–	–	24 (8-32)	12 (8-32)	–	–	–
**rBCG-N-hRSV - Uninfected**	5 (2-8)	3.33 (2-8)	–	–	22 (8-32)	18 (8-32)	–	–	–
**Unvaccinated - bRSV**	4 (2-8)	17.3 (8-32)	2.5 (2-4)	6 (2-16)	17.3 (8-32)	48 (32-64)	52 (32-64)	42 (16-64)	66 (16-128)
**rBCG-N-hRSV -bRSV**	5.3 (4-8)	85.3* (64-128)	6 (2-8)	10.6* (8-16)	26.6 (16-32)	74.6 (64-128)	60 (32-128)	34 (16-64)	53 (16-128)
**WT BCG - bRSV**	–	–	3.3 (2-8)	12.6** (8-16)	–	–	68 (32-128)	42 (16-64)	61.3 (32-128)

Virus neutralization titers measured in nasal fluid and serum on baseline (pre immune), day 0 (prior to challenge) and day 7 post infection. Two-way ANOVA followed by Sidak’s multiple comparisons test, *p<0.05, **<0.01 compared to unvaccinated - bRSV group.

## Discussion

Human RSV causes a high impact on health systems worldwide annually, being responsible for millions of hospitalizations and hundreds of thousands of deaths due to acute low respiratory tract infections in high-risk populations, which include infants, elderly and immunocompromised patients (2, 57). Despite more than five decades of research, no vaccine has been licensed to prevent RSV infection in any age group. The only prophylactic tool to prevent severe infection is Palivizumab, a humanized monoclonal antibody that is used only in high-risk infants due to its high cost (58, 59). While several vaccine strategies are under development and clinical testing for either pregnant women, infants, and the elderly (58, 60), the development of efficacious vaccines targeting specific age groups faces different challenges at both pre-clinical and clinical levels. RSV-associated morbidity and mortality are higher in infants under one-year-old in low-income countries; thus children remain a critical target population to implement therapeutic and preventive measures (57, 61). Importantly, vaccines for this age group must be able to elicit robust, long-lasting immunity in a population that is not well equipped to do so and might present significant levels of circulating MDA (60, 62, 63). Additionally, vaccines should avoid VED after natural infections, a phenomenon described after FI-RSV vaccination and subsequent natural infection in seronegative infants, that has been linked to a Th2-biased, dysregulated immune response characterized by inadequate antibody production and weak cytotoxic CD8^+^ T cell response (12, 22, 64). Those challenges underscore the importance of rational design and proper testing of each candidate.

Our group has developed a recombinant BCG vaccine expressing hRSV Nucleoprotein (24–28). This vaccine is aimed to prevent severe RSV infection in infants and is the sole vaccine in clinical development intended for use in neonates (30). Intradermal administration of 10^5^ CFU of a GMP rBCG-N-hRSV in healthy male adult volunteers is safe and well-tolerated, as demonstrated in phase I clinical trial (28). To evaluate the safety and immunogenicity of the rBCG-N-hRSV in a neonatal model of RSV infection, we tested the rBCG-N-hRSV vaccine in a neonatal calf model of bRSV infection. The neonatal calf represents a tractable model of infant immunity and a homologous model of RSV infection displaying key clinical and pathological similarities to infant RSV infection, representing a suitable model to study antiviral immunity and to develop preventive strategies, and thus, several vaccines targeting hRSV have been tested in young calves, as we recently reviewed (35). Our results from two independent studies on newborn dairy calves suggest that a dose of 10^6^ CFU of GMP rBCG-N-hRSV, administered within the first week of life and boosted 14 days after first immunization, is safe and well-tolerated (**Figure 1**). As expected, local reactions to the recombinant vaccine on the injection site were observed, including minor inflammation and swelling, similar to the lesions described in mice (25) and healthy adults (28), and BCG-vaccinated calves (65). Those reactions, which could be attributed to BCG components of the vaccine, were transient and self-resolved before five days. While those reactions were present in 11 of 12 calves during Study 1, only one calf in Study 2 showed a similar reaction. Calves vaccinated with WT BCG showed no detectable local reactions. Reactogenicity differences might be related to the different manufacturing processes of the WT and recombinant BCG vaccines, and to the outbred condition of calves. Importantly, no systemic adverse effects, such as fever, were seen in any vaccinated calf during the 28-day post-immunization period, suggesting that rBCG-N-hRSV vaccination has an adequate safety profile in neonates. Similarly, it is well known that BCG immunization of immunocompetent infants is safe, with a very low incidence of serious adverse effects (66, 67).

Calves received an aerosol challenge with ~10^4^ TCID_50_/mL of bRSV strain 375, 14 days after the booster immunization (**Figure 1**). Clinical disease was evident in most unvaccinated challenged animals starting 4 dpi, and their condition turned more severe towards day 7 pi. A rise in the clinical score (**Figures 2A, B**) and a trend in increased gross pathology (**Figures 2C, D**) were evident in unvaccinated calves but not in rBCG-N-hRSV-vaccinated animals in both studies. Starting from 4dpi clinical score of unvaccinated animals was significantly higher in comparison to rBCG-N-hRSV-vaccinated calves. Clinical disease and elevated body temperature (**Supplementary Figure 1**) were more evident in Study 1 in comparison to Study 2 (**Figures 2A, B**), with one calf being prematurely euthanized due to severe disease. For that reason, we chose to present and analyze the results from both studies separately. Similarly, while unvaccinated infected calves had increased relative neutrophil infiltration in BAL samples in comparison to uninfected controls, rBCG-N-hRSV vaccination decreased neutrophil infiltration, which is a recognized disease parameter in human and bovine RSV infection (**Figures 3C, D**) (41, 56, 68). Regarding eosinophils, which some studies had previously shown to be augmented in calves suffering VED (44, 45, 69), we observed no significant changes between vaccinated and unvaccinated animals (**Supplementary Figures 2C, G**). Since BAL composition, clinical score and gross pathology are used to evaluated whole-lung and systemic effects, our results suggest that rBCG-N-hRSV vaccination of neonatal calves with MDA protects against severe RSV infection. However, we found no differences in histopathology scores (**Figures 3A, B**), viral loads or viral shedding in nasal secretions (**Table 1**) when comparing representative lesioned lung samples from vaccinated and unvaccinated calves. These data suggest that protection would be partial and that the antiviral immunity elicited by the chosen vaccination scheme in this model is not optimally tuned to fully prevent virus replication and spread in calves. Further studies are required to define a more efficacious vaccination scheme.

A major challenge in development of RSV vaccines for infant and calf populations is to generate active immunity in presence of maternally derived antibodies (70, 71). While some studies have suggested that MDA can prevent RSV infection (72, 73), it is known that RSV severe disease can occur in presence of MDA in both calves (51, 74) and humans (75–78). Although the outcome of vaccination is shaped by multiple factors, it is well documented that MDA can interfere with generation of active immunity in vaccinated calves (79–81). Successful strategies to overcome this major hurdle include mucosal vaccination and triggering cell-mediated immune mechanisms, i.e., by adjuvanted parenteral vaccines (70, 82) The BCG vector employed in this formulation is well recognized as a highly immunogenic vaccine or adjuvant, being a potent stimulator of Th1 immunity in adults and newborns, triggering antigen-specific CD4^+^ and CD8^+^ T cells (83–85) Efficient cell-mediated immunity and IFN-γ secretion have been observed after s.c. BCG vaccination of calves as early as 8 h after birth (86), and one-week-old BCG-vaccinated calves show cellular and IFN-γ responses to PPD-B comparable to adult animals (87). The rBCG-N-hRSV vaccine elicits efficient cellular and humoral Th1 immunity against hRSV in mice, promoting the early recruitment of CD4^+^ and CD8^+^ T cells in the lung, but also a specific antibody response against several viral proteins and increased serum neutralizing activity (24–27). Here, we observed significantly increased N-hRSV and bRSV-specific CD4^+^ T cells and bRSV-specific CD8^+^ T cells in lung-draining TBLNs near the site of infection (**Figures 4B, D**), as well as increasing trends in peripheral blood of rBCG-N-hRSV-vaccinated calves at 7 dpi (Figure A,C). These proliferative responses were associated with an IFN-γ response to bRSV and PPD-B in the TBLN (**Figure 5B**) and a robust peripheral IFN-γ and IL-17 response to mycobacterial and viral antigens (**Figures 5A, C**), showing that the rBCG-N-hRSV vaccine is immunogenic in neonatal calves with MDA, inducing a Th1/Th17 cellular response to both the N-hRSV protein and bRSV, that might be suitable to overcome Th2 bias and dysbalanced cytokine responses associated with RSV infection in infants (88, 89) and calves (90–92). Although we did not determine specific T cells in the lung, detection of virus-specific cells at TBLNs suggests that specific responses took place in the lung as early as 7dpi. However, we found that neither IFN-γ nor IL-17 were upregulated in BAL at 7dpi in antigen recalls assays after viral antigen stimulation (**Supplementary Figure 4**). The ability of our candidate vaccine to recruit virus-specific T cells and their ability to impact their cytokine milieu should be addressed in future studies as the early recruitment of CD4+ and CD8+ T cells is required to elicit antiviral immunity and prevent lung damage according to our previous studies (26).

Natural RSV infection in infants generates a weak, short-lived primary IgG and IgA response that returns to pre-infection levels within less than four months (93–95). Moreover, the generation of antibody responses might be affected by circulating maternal antibodies (96, 97). Neutralizing antibodies might have an important role in the prevention of RSV infection and are an important vaccination goal (12, 98). RSV. Mucosal RSV-specific IgA has been correlated to protection in both adults (99, 100) and infants (101). In calves, IgA and IgM can be detected since eight days after bRSV challenge (102), and protection from respiratory disease has been achieved after vaccination with a mucosal polyanhydride nanovaccine by inducing significant levels of RSV-specific IgA in nasal secretion and BAL, as well as cellular responses in airways and peripheral blood (51). Here, parenteral vaccination with rBCG did not induce significant levels of IgA or NTs in nasal secretion prior to challenge, with all animals having little to no NTs. At 7dpi, significantly higher IgA levels were found in nasal secretions of rBCG-N-hRSV-vaccinated animals only Study 1 (**Figures 4A, C**), however, increased NTs were observed in nasal secretions in rBCG-N-hRSV-vaccinated animals in both studies (**Table 2**). These results indicate that parenteral rBCG-N-hRSV vaccination can induce mucosal and systemic immune responses to bRSV in neonatal calves with MDA. The enhanced NTs in nasal secretions were associated to reduced clinical disease (**Figure 2**) but not to an effect on viral shedding (**Table 1**). Although the induction of mucosal responses is generally sought by mucosal routes of immunization, several vaccines have demonstrated to induce mucosal immunity after parenteral administration (103, 104), including an adjuvanted, modified-live multivalent vaccine targeting bRSV and other bovine respiratory viruses (105). The mechanisms for these local immune priming following parenteral vaccination are not well understood (103, 104), but might be dependent on the type of vaccine, immunization route, use and type of adjuvants, and several other factors. Considering previous evidence arguing for a protective role of mucosal IgA against RSV in infants and calves, and the results from these studies, we hypothesize that aerosol or intranasal administration of the rBCG-N-hRSV vaccine could be an efficient way to induce efficient immunity against RSV by mounting both an early mucosal neutralizing response and a sustained systemic cellular response towards RSV able to prevent virus replication and induce protective memory responses.

Although our candidate vaccine encodes the nucleoprotein form hRSV, it was able to elicit N-hRSV and virus-specific cellular responses (**Figures 4** and **5**). Along these lines, the Nucleoprotein is the most conserved antigen when comparing human and bovine viral species, reaching 93% of AA identity according to previous analyses (106). It is interesting to note that although RSV nucleoprotein might not be a neutralizing target, the rBCG-N-hRSV vaccine is able to elicit antibody responses to other RSV antigens through a linked recognition mechanism, including surface antigens G and F, as previously demonstrated in mice models (27). Interestingly, WT BCG vaccinated calves also exhibited increased NTs in nasal secretions at 7dpi. This observation suggests that the increased NT seen in these animals might be related to unspecific immune priming other than a linked recognition mechanism, and suggests that the increase in IgA and NTs in calves vaccinated with rBCG-N-hRSV might be explained by both unspecific and specific effects. While some studies on human infants have shown that BCG vaccination can impact heterologous antibody production (107, 108), conflicting evidence indicates that timing of vaccination and several other factors might impact on such responses, as discussed previously (109).

When measuring bRSV-specific serum IgG and NT, an increasing trend in rBCG-N-hRSV-vaccinated calves at 7dpi was observed in Study 1. Considering that result, for Study 2 we determined serum bRSV-specific IgG1 and IgG2 levels, which display different kinetics in peripheral blood and are associated with different T helper phenotypes (35, 43). IgG1 levels were higher at baseline and then decreased towards day 28 post-immunization, suggesting that rBCG vaccination did not induce significant peripheral IgG1 levels. Regarding Ig2, comparisons between baseline and 7dpi levels showed a decreasing trend in unvaccinated WT BCG groups but not in rBCG-N-hRSV-vaccinated animals, which showed a modest, although significantly higher level of virus-specific IgG2 at 7dpi when compared to unvaccinated animals. This suggests that vaccination with rBCG favors an IgG2 response in peripheral blood of neonatal calves, which is consistent with a Th1 phenotype in bovines and with our previous results (24, 35). Despite that difference, serum NTs from study 2 showed similar kinetics regardless of the vaccination status, with similar levels at baseline, which tended to drop before the challenge and then to slightly rise at 7dpi, without differences between groups. Since all animals received colostrum after birth, it is no surprise to find NTs as high as 128 and the higher IgG1 levels at baseline. Although baseline NTs were not measured during Study 1, it is possible that those NTs might account for the less severe disease in Study 2 calves in comparison to Study 1. The use of colostrum-replete animals allows us to test vaccine candidates in the most physiological model that resembles the scenario of vaccinating infants with MDA. More importantly, our results indicate that vaccination with rBCG-N-hRSV is immunogenic in neonatal calves even in the presence of significant virus-specific circulating antibodies. Further studies should address the duration and role of cellular and humoral responses elicited by vaccination and infection.

Previous studies evaluating the efficacy of rBCG-N-hRSV and rBCG-P-hMPV vaccines against hRSV and human Metapneumovirus infection in mice, respectively, have shown reduced disease parameters in WT BCG vaccinated control animals (27, 110), which might be explained by unspecific functional effects of BCG vaccination on innate immune cells (111). Although not evaluated here, *in vitro* and *in vivo* innate training has been reported in the bovine species after administration of heat-killed *M. bovis* (112) and BCG (113), and the immunomodulatory effects of BCG administration on innate cells have been numerously reported on humans (114–118). Here, WT BCG vaccination prevented a rise in the clinical score (**Figure 2B**) and slightly reduced relative neutrophil infiltration (**Figure 3D**), without modulating lung viral loads or viral shedding. As expected, WT BCG was not associated with N-hRSV or bRSV-specific CD4+ and CD8+ T cell responses in TBLNs (**Figures 4B, D**). On the contrary, both vaccines increased NTs in nasal secretions (**Table 2**). This data suggests that BCG-related unspecific immune mechanisms might confer some degree of heterologous protection against bRSV challenge. Concurrently, increased relative macrophage frequencies were seen in WT BCG and rBCG-N-hRSV-vaccinated animals (Supp Fig2 A,E), indicating that vaccination significantly modulated BAL innate cellular composition. While unspecific effects of BCG or recombinant BCG vaccines might be beneficial against some infectious diseases (114–116, 119–121), and thus might be tested as potential immunomodulatory strategies for in infants and young animals, the extent of these effects and the impact on the induction of adaptive immunity should be comprehensively studied in order to fine-tune cellular protective mechanisms on candidate vaccines.

In summary, a two-dose subcutaneous administration of 10^6^ CFU of GMP rBCG-N-hRSV is safe and well-tolerated in neonatal calves with MDA, inducing mucosal humoral immunity and systemic cellular immunity against bovine RSV, skewed towards a Th1 phenotype. Besides, vaccination conferred partial protection to bRSV, reducing clinical disease severity and modulating neutrophil infiltration in the lower respiratory tract, without sings of enhanced disease. These results support further investigation on the use of the candidate vaccine for prevention of RSV in infants and calves.

## Data Availability Statement

The raw data supporting the conclusions of this article will be made available by the authors, without undue reservation.

## Ethics Statement

The animal study was reviewed and approved by Iowa State University Institutional Animal Care and Use Committee (IACUC-18-232) and Institutional Biosafety Committee (IBC-18-076).

## Author Contributions

Conceived and designed the experiments: JM, AK, FD, and MG-M. Performed the experiments: JM, FD, MG-M, and PM. Analyzed the data: FD, JM, MG-M, DR-P, and AK. Contributed reagents/materials/analysis tools: AK and JM. Wrote the paper draft: FD. Edited the paper: FD, JM, DR-P, and AK. All authors contributed to the article and approved the submitted version.

## Funding

This work was funded by Millennium Institute on Immunology and Immunotherapy, Programa ICM-ANID, ICN09_016 (to AK) and Fondo de Fomento al Desarrollo Científico y Tecnológico- D11E1080 (to AK), and the Agencia Nacional de Investigación y Desarrollo (ANID) (doctorate grant N-21170620 to FD). Funding was also provided by the National Institutes of Health, National Institute of Child Health and Human Development Award R01HD095880 and United States Department of Agriculture, National Institute of Food and Agriculture Award 2020-67015 (to JM). AK is a Helen C. Levitt Visiting Professor at the Department of Microbiology and Immunology of the University of Iowa. Funders had no role in study design, data collection and analysis, decision to publish, or preparation of the manuscript.

## Conflict of Interest

A patent for the rBCG-N-hRSV vaccine has been filled and issued by Pontificia Universidad Catolica de Chile in several countries.
