# Supplementary material for: A Recombinant BCG Vaccine Is Safe and Immunogenic in Neonatal Calves and Reduces the Clinical Disease Caused by the Respiratory Syncytial Virus
Source: Front Immunol. 2021 Apr 26;12:664212. doi: 10.3389/fimmu.2021.664212 (PMC8108697; doi:10.3389/fimmu.2021.664212)
Supplement: Supplementary file 1 [file DataSheet_1.pdf]

Supplementary Material

Group	Study 1				Study 2			
	Negative	Suspect	Positive	Total	Negative	Suspect	Positive	Total
**Unvaccinated**	8	0	0	8	8	0	0	8
**WT BCG**	--	--	--	-	6	0	2	8
**rBCG-N-hRSV**	4	2	5	11	2	2	4	8

Supplementary table 1. **Summary of Comparative Cervical Test Results**. Newborn calves were vaccinated with rBCG-N-RSV (Studies 1 and 2) or WT BCG (Study 2) and boosted 14 days after prime immunization. Then, 0.1 mL (1 mg/mL concentration) of *M. avium* PPD-B and of *M. bovis* PPD-B were injected in the neck skin of calves. Local reactions were registered as increase of skin thickness for both injection sites, 72h after antigen inoculation and before bRSV challenge. Negative: No reaction or no increase of PPD-B over PPD-A; Suspect: Increase of 1-3mm of PPD-B over PPD-A; Positive: Increase ≥ 4mm of PPD-B over PPD-A.

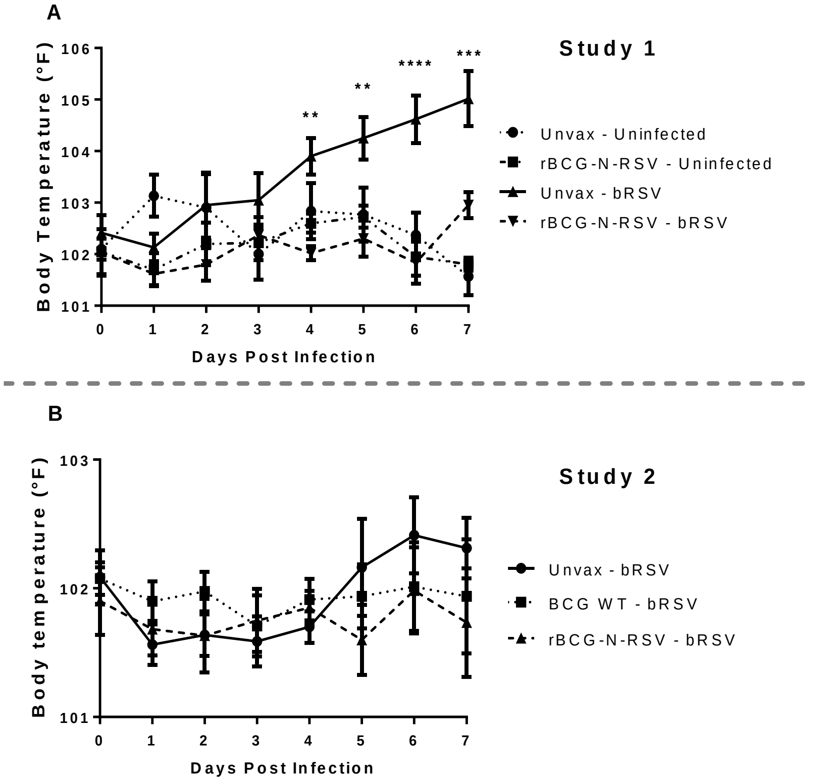

Supp Figure 1. rBCG-N-RSV vaccination prevents fever in neonatal calves. Newborn calves were vaccinated with rBCG-N-RSV (Studies 1 and 2) or WT BCG (Study 2) and boosted 14 days after prime immunization. Fourteen days after the booster, calves were infected with bRSV strain 375 via aerosol inoculation. Body temperatures in F° for **(A)** Study 1 and **(B)** Study 2. Calves in all four groups were monitored daily by a blinded observer and assigned a clinical score using the criteria outlined in Materials and Methods. Data represented as mean ± SEM. *p<0.05 **p<0.01 ***p<0.001 ****p<0.0001 as determined by 2-way ANOVA with repeated measures and Sidak's multiple comparisons test.

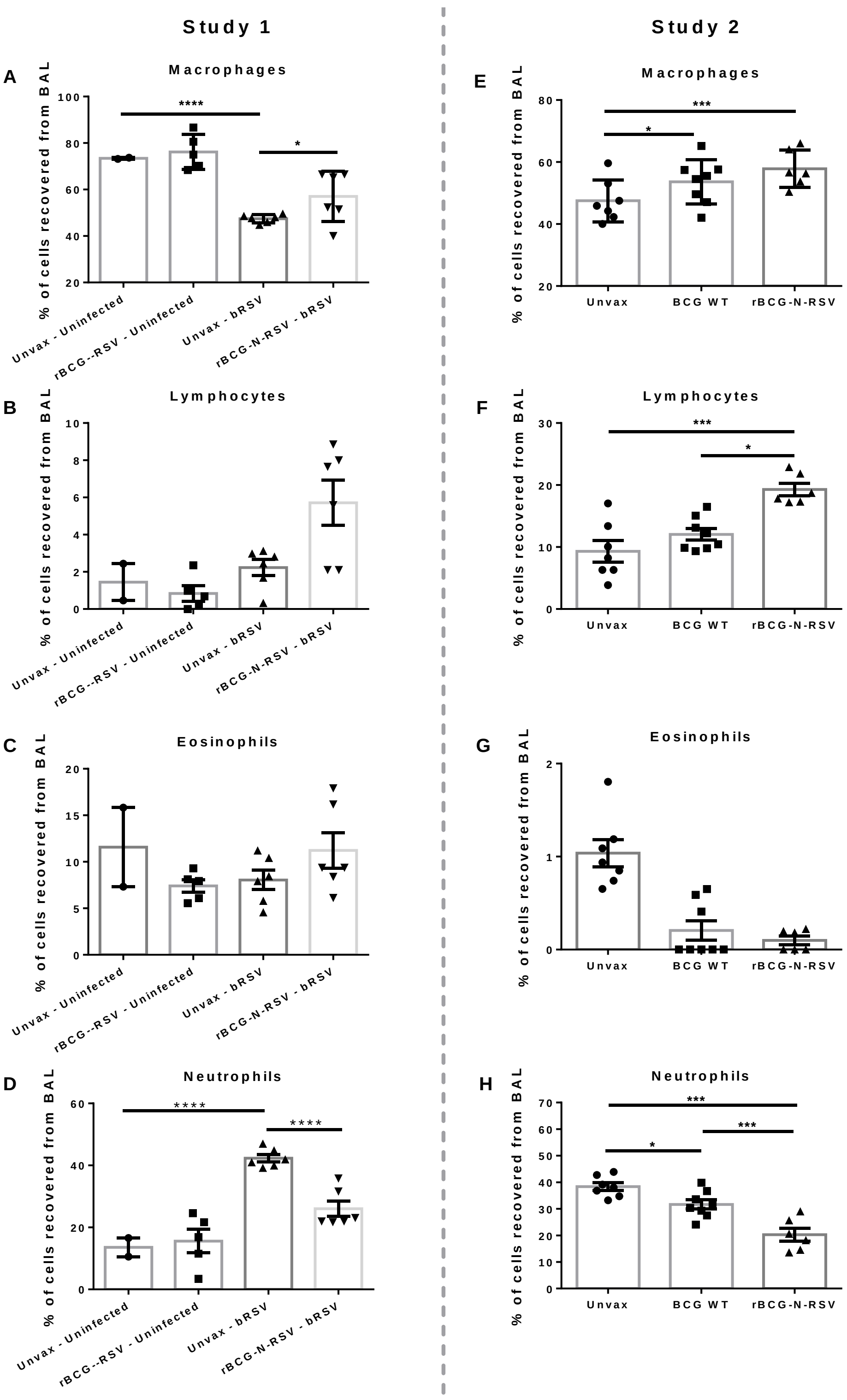

Supp. Figure 2. rBCG-N-RSV vaccination modifies BAL cells relative frequency after bRSV infection. On day 7 post-infection, BAL samples were collected, and cytospins prepared. The cells were differentially stained with Modified Wright stain. The number of neutrophils, macrophages, lymphocytes, and eosinophils were determined by microscopy.

Data are depicted as mean relative frequencies of each population ± SEM. *p<0.05 ***p<0.001 ****p<0.0001 as determined by 2-way ANOVA and Sidak's multiple comparisons test.

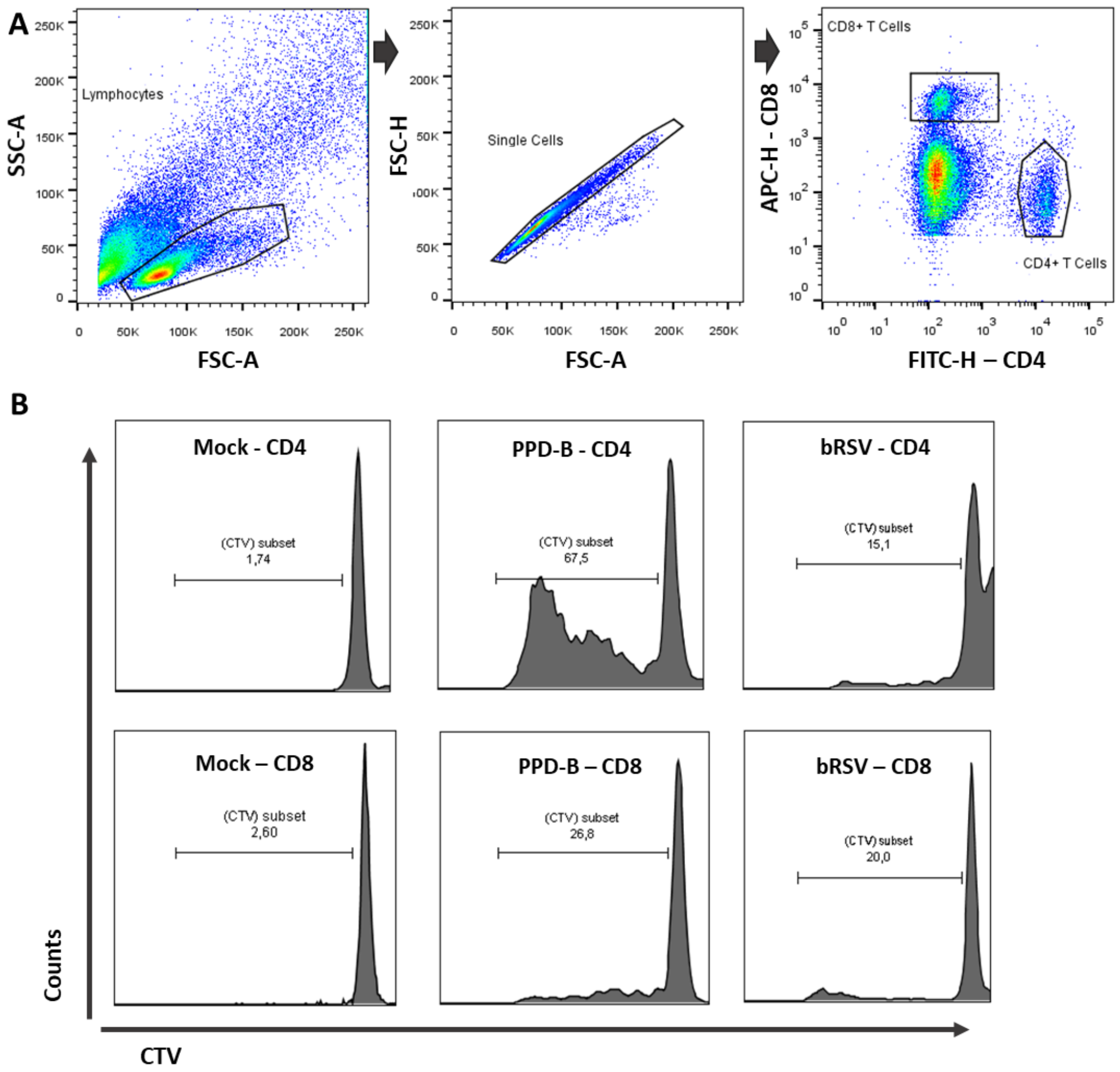

Supp. Figure 3. Representative gating plots for identification of dividing CD4+ and CD8+ T cells. Study 1 PBMCs and Study 2 Tracheobronchial lymph node cells (TBLNs) were isolated on day 7 after infection, labeled with Cell Trace Violet, restimulated in vitro as in Figure 5, and analyzed by flow cytometry for determining dividing CD4+ and CD8+ cells. **(A)** Gating hierarchy as depicted by arrows: lymphocytes (first gate), single Cells (second gate), and CD4+ and CD8+ cells (third gate). **(B)** Determination of dividing subsets: Results from each stimulation condition were calculated as change of CTV subset over mock. Representative samples of mock, PPD-B and bRSV conditions from a rBCG-N-RSV-vaccinated, bRSV-infected animal are shown.

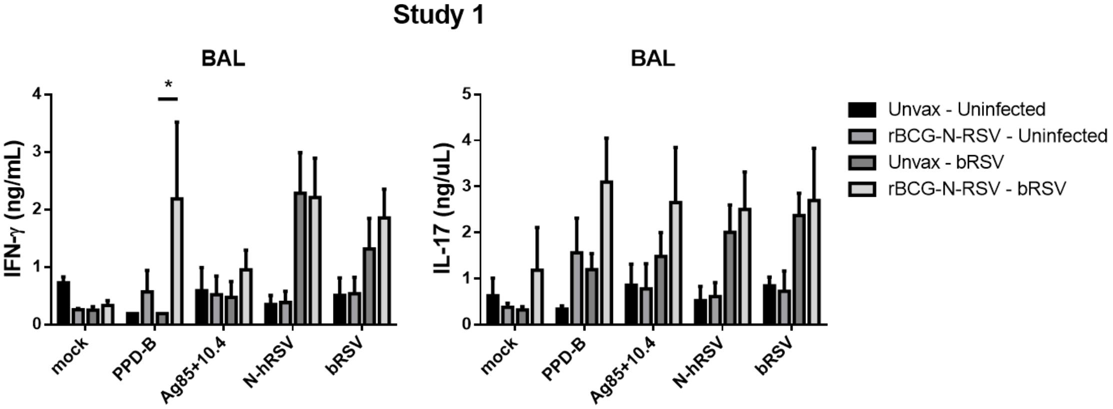

Supp. Figure 4. **Increased virus-specific cytokine production by PBMCs from rBCG-N-RSV vaccinated animals**. On day 7 post infection, BAL samples were collected and stimulated with PPD-B, Ag85A/TB10.4, N-hRSV or bRSV for 72 hours. Cell culture supernatants were analyzed for concentrations of bovine IFN-γ and IL-17A. *$p<0.05$ as determined by 2-way ANOVA and Sidak's multiple comparisons test.
